# Tissue microarray analysis of human *FRAT1* expression and its correlation with the subcellular localisation of *β*-catenin in ovarian tumours

**DOI:** 10.1038/sj.bjc.6602988

**Published:** 2006-02-14

**Authors:** Y Wang, S M Hewitt, S Liu, X Zhou, H Zhu, C Zhou, G Zhang, L Quan, J Bai, N Xu

**Affiliations:** 1 Laboratory of Cell and Molecular Biology, Cancer Institute & Cancer Hospital, Chinese Academy of Medical Sciences & Peking Union Medical College, Beijing 100021, People's Republic of China; 2 Tissue Array Research Program, Laboratory of Pathology, Center for Cancer Research, National Cancer Institute, National Institutes of Health, Bethesda, MD 20892-4605, USA

**Keywords:** FRAT1, *β*-catenin, Wnt pathway, ovarian cancer, tissue microarray

## Abstract

The mechanisms involved in the pathogenesis of ovarian cancer are poorly understood, but evidence suggests that aberrant activation of Wnt/*β*-catenin signalling pathway plays a significant role in this malignancy. However, the molecular defects that contribute to the activation of this pathway have not been elucidated. Frequently rearranged in advanced T-cell lymphomas-1 (FRAT1) is a candidate for the regulation of cytoplasmic *β*-catenin. In this study, we developed *in situ* hybridisation probes to evaluate the presence of *FRAT1* and used an anti-*β*-catenin antibody to evaluate by immunohistochemistry the expression levels and subcellular localisation of *β*-catenin in ovarian cancer tissue microarrays. Expression of *FRAT1* was found in some human normal tissues and 47% of ovarian adenocarcinomas. A total of 46% of ovarian serous adenocarcinomas were positive for *FRAT1* expression. Accumulation of *β*-catenin in the nucleus and/or cytoplasm was observed in 55% ovarian adenocarcinomas and in 59% of serous adenocarcinomas. A significant association was observed in ovarian serous adenocarcinomas between *FRAT1* and *β*-catenin expression (*P*<0.01). These findings support that Wnt/*β*-catenin signalling may be aberrantly activated through *FRAT1* overexpression in ovarian serous adenocarcinomas. The mechanism behind the overexpression of *FRAT1* in ovarian serous adenocarcinomas and its significance is yet to be investigated.

Ovarian cancer is the sixth most common cancer in women worldwide and a highly aggressive gynaecological malignancy producing almost 125 000 deaths yearly (Parkin *et al*, 2005). Despite advances in detection and cytotoxic therapies, only 30% of patients with advanced-stage ovarian cancer survive 5 years after initial diagnosis (Parkin *et al*, 2005). Therefore, an understanding of the molecular mechanisms involved in ovarian cancer pathogenesis and progression has the potential to have a significant impact on the outcomes for this devastating disease. Most ovarian neoplasms are adenocarcinomas that occur as four major histological subtypes, serous, mucinous, endometrioid and clear cell, with serous being the most common. Current data indicate that each of these histological subtypes is associated with distinct morphologic and molecular genetic alterations (Bell, 2005). Further investigations of the molecular mechanisms promoting ovarian cancer are necessary to determine how each of the subtypes emerge. Relatively little is known about the molecular events that lead to the development of ovarian cancer.

Recently, accumulation of nuclear and/or cytoplasmic *β*-catenin has been observed in ovarian cancer (Gamallo *et al*, 1999; Wright *et al*, 1999; Rask *et al*, 2003; Kildal *et al*, 2005). *β*-Catenin is a multifunctional protein involved in at least two important biological processes: cell–cell adhesion and Wnt/*β*-catenin signalling pathway (Nelson and Nusse, 2004). It is believed that accumulation of *β*-catenin in the cytoplasm favours its translocation to the nucleus as a cofactor for transcription factors of the T-cell factor/lymphoid enhancing factor (TCF/LEF) family and activates the transcription of Wnt/*β*-catenin target genes, which are involved in cellular differentiation and proliferation (Giles *et al*, 2003). Although compelling evidence has indicated a critical role for signalling by *β*-catenin in the tumorigenesis of ovarian cancer, mutations in the *β-catenin* gene are infrequent in ovarian carcinoma and interestingly only described in the endometrioid type of epithelial ovarian tumours (Gamallo *et al*, 1999; Wright *et al*, 1999; Ueda *et al*, 2001; Giles *et al*, 2003). Mutations of *adenomatous polyposis coli* (*APC*) or *Axin* occur rarely in ovarian cancer (Giles *et al*, 2003). Therefore, additional mechanisms can be used to upregulate *β*-catenin levels in other types of ovarian tumours. Previous studies have shown that frequently rearranged in advanced T-cell lymphomas-1 (FRAT1) is strikingly overexpressed in some human cancer cells (Saitoh and Katoh, 2001; Saitoh *et al*, 2002). Furthermore, FRAT1 is a positive regulator of the Wnt/*β*-catenin pathway, which can inhibit glycogen synthase kinase 3 (GSK-3) activity towards *β*-catenin, at least in part, by preventing Axin binding to GSK-3 (Ferkey and Kimelman, 2002; Fraser *et al*, 2002; Giles *et al*, 2003; van Amerongen and Berns, 2005). However, whereas FRAT1 is a candidate for the regulation of cytoplasmic *β*-catenin, little is known with regard to the molecular relationship between FRAT1 and *β*-catenin in ovarian cancer.

In this study, we developed *in situ* hybridisation probes to evaluate the presence of *FRAT1* and used an anti-*β*-catenin antibody to evaluate by immunohistochemistry the expression levels and subcellular localisation of *β*-catenin in formalin-fixed, paraffin-embedded human normal tissues and ovarian tumour tissues. This study was also aided by the use of tissue microarrays, allowing a much larger cohort of patient samples to be evaluated in a more standardised and rapid fashion. We demonstrate here that *FRAT1* is expressed in some human normal tissues and overexpressed in human ovarian cancer. There is an obvious correlation between *FRAT1* overexpression and aberrant nuclear and/or cytoplasmic accumulation of *β*-catenin in serous adenocarcinoma of ovary. Wnt/*β*-catenin signalling may be aberrantly activated through *FRAT1* overexpression in ovarian serous adenocarcinomas.

## MATERIALS AND METHODS

### Construction of tissue arrays

TARP5-T-BO-1 tumour tissue arrays were constructed by the NCI TARP Lab using anonymous donor blocks obtained from the Cooperative Human Tissue Network. Detailed information about TARP5-T-BO-1 tumour tissue arrays is available at www.cancer.gov/tarp. The arrays were produced as described previously (Hewitt, 2004), using a manual tissue arrayer (Beecher Instruments, Silver Spring). The array design included specimens of 75 ovarian malignancies of surface epithelial origin, 65 breast carcinomas and 35 cores of normal tissues representing 17 different histologies. Sections (5 *μ*m) were cut from the array blocks using tape sectioning materials from Instrumedics (Hackensack, NJ, USA).

### Plasmids construction

The FRAT1 expression vector was constructed by cloning the open reading frame of the human *FRAT1* cDNA obtained from HEK293 cells into pcDNA3 vector (Invitrogen, CA, USA). Correct cloning of *FRAT1* cDNA fragments was confirmed by sequencing. The resulting plasmid was designated FRAT1*/*pcDNA3. Probes for *in situ* hybridisation were generated by *in vitro* transcription using SP6 polymerase (Roche, Germany) as described below.

### *In situ* hybridisation

For *in situ* hybridisation, digoxigenine (DIG)-labelled *FRAT1* probes were prepared using a DIG-RNA-labelling kit (Roche), according to the manufacturer's instructions. In brief, FRAT1*/*pcDNA3 plasmids were linearised with *Hin*dIII and DIG-labelled probes were generated by *in vitro* transcription using SP6 polymerase (Roche). The amount of transcripts was monitored by 2% agarose gel electrophoresis and determined by spectrophotometry. Labelling efficiency was controlled by dot blot analysis of serial probe dilutions.

*In situ* hybridisation was performed according to the method described previously (Zhou *et al*, 2005). In brief, tissue sections were deparaffinised, rehydrated in serial dilutions of ethanol, and postfixed in 4% Tris-buffered saline (TBS)-buffered paraformaldehyde. Samples were treated with 0.2 mol l^−1^ HCl for 10 min at room temperature and then permeabilised using proteinase K (100 mg l^−1^) at 37°C for 15 min. Digestion was stopped by washing the samples in phosphate-buffered saline (PBS) (pH 7.4). The samples were then dehydrated in serial dilutions of ethanol. DIG-labelled *FRAT1* probes were diluted in hybridisation buffer (50% formamide, 4 × SSC, 5% dextran sulphate, 5 × Denhardt's solution and 200 mg ml^−1^ denatured salmon sperm DNA). After probes were applied, the samples were covered with sterile coverslips. Hybridisation was performed overnight at 42°C in a sealed humidified chamber containing 50% formamide. Nonspecific binding or unbound probes were removed by the following posthybridisation washes: 2 × SSC at room temperature (2 × 10 min), 1 × SSC at room temperature (2 × 10 min), and finally, the sections were washed in TBS containing 0.1% Tween-20 (TTBS). Hybridisation signals were detected using an alkaline phosphatase (AP)-conjugated anti-DIG antibody (Roche). After washing in TTBS, the slides were incubated with nitro blue tetrazolium/5-bromo-4-chloroindol-3-yl phosphate (NBT/BCIP) (Roche) for 10 min. The *FRAT1*-positive cells were purple–blue stained. The negative controls were incubated with hybridising solution without *FRAT1* probes.

### Immunohistochemistry

Immunohistochemistry was performed using an UltraSensitive™ Kit (Maixin-Bio) according to the manufacturer's protocol as described previously (Wang *et al*, 2005). In brief, tissue sections were deparaffinised, and rehydrated in serial dilutions of ethanol. Endogenous peroxidase activity was blocked with 10-min incubation in a 3% hydrogen peroxide in PBS. Antigen retrieval was performed by boiling in citrate buffer (0.01 M, pH=6.0) in a microwave oven. To reduce background nonspecific staining, slides were incubated with normal nonimmune serum for 30 min. The excess serum was removed and slides were incubated for 60 min with anti-*β*-catenin (C19220; 1 : 50; Transduction Lab, KY, USA). Each incubation step was carried out at room temperature and was followed by three sequential washes (5 min each) in PBS. Slides were then incubated with biotin-conjugated second antibody for 30 min, followed by incubation with streptavidin–peroxidase for 30 min. Peroxidase activity was identified by applying 3,3′-diaminobenzidine tetrachloride (DAB). Sections were rinsed in water and counterstained with haematoxylin. Finally, slides were dehydrated in alcohol, cleared in xylene. Slides were also stained in the absence of primary antibody to evaluate nonspecific secondary antibody reactions.

### Evaluation of staining

All *in situ* hybridisation and immunostaining experiments were assessed by two experienced pathologists who were blinded to the origin of the sections. The membranous, cytoplasmic and nuclear staining was determined separately for each specimen. The staining intensity was graded as follows: 0, no staining; 1, weak staining; 2, moderate staining and 3, intense staining. Owing to too few cells or core missing in some sample cores, only 60 ovarian samples out of 75 cores and 12 different normal tissue samples in this tissue microarray were interpretable. For specimens that were uninterpretable, a score of NA was given. The data from breast samples are not shown here. In each case, the staining was scored as an average throughout the spot. *FRAT1* expression within the tumour tissue was evaluated and categorised according to the staining intensity. Tumours were then further grouped into low (score 0) and high (scores 1–3) expression of *FRAT1*. Meanwhile, *β*-catenin protein expression profiles in cancer tissues were further grouped into cytoplasm/nucleus accumulation or no accumulation.

### Statistical analysis

SPSS for Windows (SPSS Inc.) was used for statistical analysis. Correlation between the *FRAT1* expression levels and *β*-catenin expression profiles on a per case basis was further analysed by Spearman's *ρ* correlation coefficient test. Values of *P*<0.05 were considered statistically significant.

## RESULTS

### Site of reaction products

**Figure 1:**
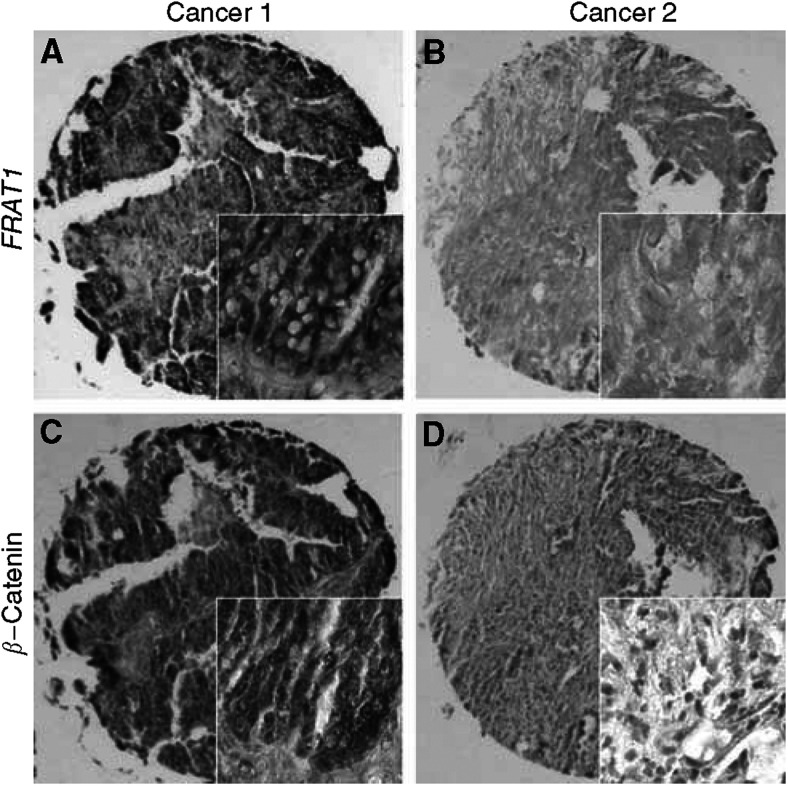
Correlation between *FRAT1* overexpression and *β*-catenin localisation in human serous adenocarcinomas of ovary. Tissue microarrays of paraffin-embedded ovarian adenocarcinomas were stained with *in situ* hybridisation probes against *FRAT1* or anti-*β*-catenin antibody and visualised by NBT/BCIP or DAB staining, respectively. Left panels, a representative high-*FRAT1* staining specimen (**A**) with accumulation of *β*-catenin in the cytoplasm and nucleus (**C**); right panels, a representative specimen with low-*FRAT1* staining (**B**) with no accumulation of *β*-catenin (**D**). Figures are × 100, and inserts are × 400.

The product of *in situ* hybridisation with the probes directed at *FRAT1* was located in the cytoplasm of the cells (Figure 1A). Meanwhile, *β*-catenin immunostaining demonstrated two distinct staining patterns: one that was predominantly cytoplasmic, sometimes with concomitant nuclear staining and reduced membranous staining (Figure 1C); and one that was with no cytoplasmic or nuclear staining, often with membranous staining (Figure 1D).

### Expression of *FRAT1* in human normal tissues

**Figure 2:**
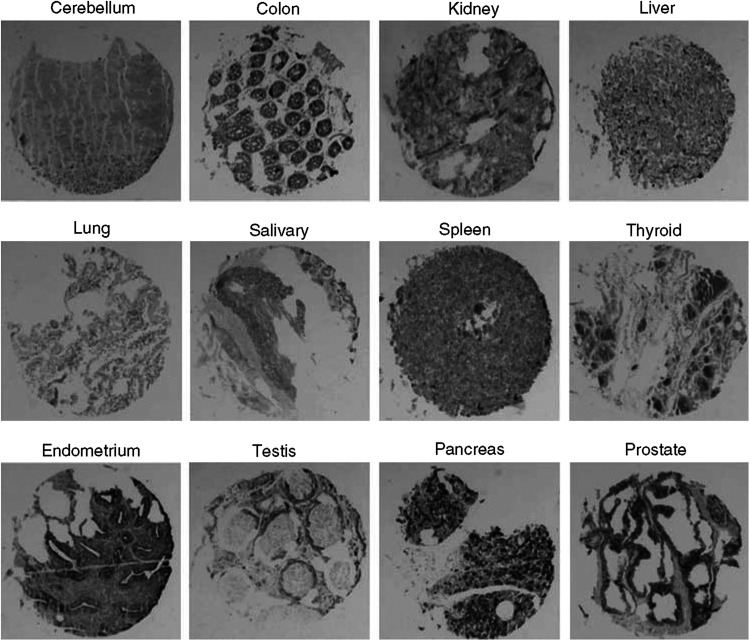
Expression of *FRAT1* in human normal tissues. The tissue microarrays containing various normal tissues were hybridised with human DIG-labelled *FRAT1* probes. Expression of *FRAT1* was not detected in human cerebellum, colon, kidney, liver, lung, salivary, spleen and thyroid; meanwhile, there was *FRAT1* expression in human endometrium, testis, pancreas and prostate.

The tissue microarrays containing various normal tissues and tumour tissues were hybridised with human DIG-labelled *FRAT1* probes. The probes span the C-terminal coding region, minimising the potential for crossreactivity between human *FRAT1* and *FRAT2*. Analysis of the tissue microarrays revealed that *FRAT1* was expressed in human endometrium, testis, pancreas and prostate (Figure 2). Expression of *FRAT1* in other human normal tissues, including cerebellum, colon, kidney, liver, lung, salivary, spleen and thyroid, was not detected (Figure 2). These results were consistent with a previous report (Freemantle *et al*, 2002).

### Expression of *FRAT1* and *β*-catenin in human ovarian tumour tissues

*FRAT1* was found in 28 of 60 (46%) ovarian adenocarcinomas investigated. In total, 46% (19 out of 41) of all ovarian serous adenocarcinomas were positive for *FRAT1* expression, followed by four out of 11 clear cell, four out of five endometrioid and one out of three mucinous adenocarcinomas (Table 1). All analysed ovarian tumours showed membrane *β*-catenin expression with variable extension and intensity. Accumulation of *β*-catenin in the nucleus and/or cytoplasm was observed in 33 of 60 (55%) ovarian adenocarcinomas. In serous adenocarcinomas, the percentage of cases with *β*-catenin accumulation was approximately 59%. Nuclear and/or cytoplasmic expression of *β*-catenin was also observed in four out of 11 clear cell, five out of five endometrioid and zero out of three mucinous adenocarcinomas (Table 1).

### Correlation between subcellular localisation of *β*-catenin and overexpression of *FRAT1* in ovarian serous adenocarcinomas

To examine whether *FRAT1* expression is correlated with increased expression and cytoplasmic/nuclear localisation of *β*-catenin in primary ovarian serous adenocarcinoma tissues, we compare the expression of *FRAT1* and the subcellular localisation of *β*-catenin in 41 primary human ovarian serous adenocarcinoma tissues by *in situ* hybridisation and immunohistochemical staining, respectively. Previous studies demonstrated that *β*-catenin has an aberrant expression pattern in ovarian serous adenocarcinomas (Rask *et al*, 2003; Kildal *et al*, 2005). Approximately 59% (24 out of 41) of ovarian serous adenocarcinoma samples showed cytoplasmic/nuclear accumulation accompanied by reduction of *β*-catenin localisation at membranes. More importantly, in most cases, *β*-catenin accumulation was correlated with high levels of *FRAT1* expression in tumour specimens, whereas tumour tissues showing no accumulation of *β*-catenin generally contained relatively low levels of *FRAT1* (Figure 1; Table 2). In 41 ovarian serous adenocarcinoma tissues examined, there was a significant correlation between *FRAT1* and *β*-catenin expression, as determined by the Spearman rank correlation test (*P*<0.01). These results suggest that *FRAT1* may modulate the Wnt/*β*-catenin pathway in ovarian serous adenocarcinomas.

## DISCUSSION

Ovarian cancer is one of the most complex malignancies with a diversity of histological subtypes, including serous, clear cell, mucinous and endometrioid (Bell, 2005). Limited information is available as for the molecular alterations in ovarian cancer. The reports about the expression of *β*-catenin in ovarian cancer are a little confusing. However, it is consistent that nuclear *β*-catenin is almost exclusively present in endometriod type of ovarian cancer (Gamallo *et al*, 1999; Wright *et al*, 1999; Kildal *et al*, 2005). In other types of ovarian cancer, including serous type, the predominant aberrant expression pattern of *β*-catenin was cytoplasmic staining without nuclear accumulation (Rask *et al*, 2003; Kildal *et al*, 2005). These confusing phenomena may indicate and also may be due to distinct molecular genetic alterations in different types of ovarian caner (Bell, 2005). Although accumulation of cytoplasmic and nuclear *β*-catenin has been observed in serous ovarian cancer (Rask *et al*, 2003; Kildal *et al*, 2005), mutations of *β-catenin* in this subtype occur rarely (Gamallo *et al*, 1999; Wright *et al*, 1999; Ueda *et al*, 2001; Giles *et al*, 2003). The mechanism of accumulation of *β*-catenin in ovarian serous adenocarcinomas might be independent of genetic alterations of the *β-catenin* as shown in other tumours (Giles *et al*, 2003). Further mechanisms to stabilise *β*-catenin protein seem to be hidden in this subtype of ovarian cancer. It has been suggested that downregulated expression of APC (Karbova *et al*, 2002) or *secreted frizzled-related proteins* (*SFRPs*) inactivation by methylation (Takada *et al*, 2004) could explain, at least partially, why ovarian serous adenocarcinomas show increased *β*-catenin protein expression in spite of the absence of gene mutation. However, none of these studies has shown any statistical correlation between these molecules and subcellular localisation of *β*-catenin in tissue samples (Karbova *et al*, 2002; Takada *et al*, 2004). Whether deregulation of these molecules contributes to the accumulation of *β*-catenin in ovarian serous adenocarcinomas is yet to be established.

*FRAT1* gene, mapping to human chromosome 10q24.1, is a human homologue of mouse proto-oncogene *Frat1* (Jonkers *et al*, 1997; Saitoh and Katoh, 2001). *Frat1* was initially cloned in a screen to identify genes that can accelerate lymphomagenesis in Moloney murine leukaemia virus (M-MuLV)-infected Eì-*Pim1* or H2-K-*Myc* transgenic mice (Jonkers *et al*, 1997). Its biological function remained unknown until its *Xenopus* homologue GSK-3-binding protein (GBP) was identified as a GSK-3-binding protein (Yost *et al*, 1998). GSK-3-binding protein is required to specify the dorsal–ventral axis in *Xenopus* embryos. It appears to inhibit the GSK-3-mediated phosphorylation of *β*-catenin, stabilising it on the future dorsal side of the embryo. Previous studies indicated that FRAT1 is a component of the Wnt signalling pathway (Yost *et al*, 1998; Thomas *et al*, 1999). Overexpression of FRAT1 leads to *β*-catenin stabilisation through dissociation of GSK-3*β* from Axin and inhibition of *β*-catenin phosphorylation (Li *et al*, 1999; Thomas *et al*, 1999). Unphosphorylated *β*-catenin is stabilised and translocated to the nucleus to associate with TCF/LEF transcription factors (Giles *et al*, 2003). The *β*-catenin/TCF complex activates the transcription of Wnt target genes such as *c-myc* (He *et al*, 1998), *cyclin D1* (Tetsu and McCormick, 1999) and human *pituitary tumour transforming gene* (*hPTTG*) (Zhou *et al*, 2005). These genes play an important role in the development and formation of some neoplasias. Thus, FRAT1 is a positive regulator of the Wnt/*β*-catenin pathway. Since the identification of human *FRAT1*, an additional homologue has been discovered in the human genome. *FRAT2*, just like *FRAT1*, functions as positive regulator of the Wnt pathway. *FRAT1* and *FRAT2* are more homologous in the N-terminal region, but are completely divergent in the C-terminal region (Saitoh *et al*, 2001; Freemantle *et al*, 2002). So our probes against human *FRAT1* span the C-terminal coding region of this gene, minimising the potential for crossreactivity between human *FRAT1* and *FRAT2*. Recently, results from van Amerongen *et al* (2005) and van Amerongen and Berns (2005) demonstrate that murine Frat is not an essential component of the canonical Wnt pathway in mice. However, since that the regulatory mechanisms about Wnt pathway are complex and still obscure, there may be the presence of parallel pathways that are functionally redundant and may compensate for the loss of Frat. Furthermore, it is notable that some previous reports suggest that Frat plays a role in tumour progression (Jonkers *et al*, 1997, 1999). Therefore, it implies that in abnormal conditions, such as in the FRAT-overexpressed cancer cells, FRAT may be an important molecular event that might lead to the aberrant activation of Wnt pathway.

In this study, we used *in situ* hybridisation probes to demonstrate the presence of *FRAT1* in paraffin-embedded human normal tissues and ovarian cancer tissues. To our knowledge, no other research group has been able to produce *in situ* hybridisation probes against *FRAT1* to date. *In situ* hybridisation seems to be a more selective and more specific technique than immunohistochemistry, but requires greater care in preparing nuclease-free solutions. Tissue microarrays were also used for the rapid and efficient analysis of large numbers of paraffin-embedded tissues (Braunschweig *et al*, 2004, 2005). By using *in situ* hybridisation to detect the expression of *FRAT1* and by using immunohistochemistry to detect the expression of *β*-catenin on tissue microarrays of paraffin-embedded ovarian adenocarcinomas, we report here that cytoplasmic/nuclear accumulation of *β*-catenin is closely associated with *FRAT1* overexpression in ovarian serous adenocarcinomas. Although it is uncertain whether FRAT1 expression is the cause or effect of cytoplasmic*β*-catenin in certain circumstances, until now, almost all data shown in the literatures indicate that FRAT1 is a positive regulator of the Wnt/*β*-catenin pathway. No reports now suggest that Wnt/*β*-catenin pathway can regulate the expression of FRAT1. Thus, a strong correlation between expression of *FRAT1* and the subcellular localisation of *β*-catenin indicates that FRAT1 may modulate the Wnt/*β*-catenin pathway in ovarian serous adenocarcinomas. A study with larger cohort seems mandatory to clarify whether the same role of FRAT1 exists in other histological subtypes of ovarian cancer, such as clear cell, endometrioid and mucinous adenocarcinomas. In addition, we report that *FRAT1* is detected in some normal human tissues, including endometrium, testis, pancreas and prostate. Similar results have been obtained by other researchers (Freemantle *et al*, 2002). Unfortunately, normal human ovary tissues are not included in this tissue microarray. However, data from Freemantle *et al* (2002) demonstrated that human *FRAT1* expression levels in the ovary were relatively low but detectable. van Amerongen and Berns (2005) suggest that murine *Frat* is expressed in rapidly dividing cells (van Amerongen *et al*, 2005). Similar expression pattern of human *FRAT1* may also be observed, but it needs further investigation.

In conclusion, *FRAT1* mRNA and *β*-catenin protein expression were studied in 60 ovarian adenocarcinomas, including 41 serous, 11 clear cell, five endometrioid and three mucinous adenocarcinomas. We report the expression pattern of *FRAT1* in some normal human tissues and that overexpression of *FRAT1* in ovarian serous adenocarcinomas is significantly associated with cytoplasmic/nuclear accumulation of *β*-catenin. Wnt/*β*-catenin signalling may be aberrantly activated through *FRAT1* overexpression in ovarian serous adenocarcinomas. The mechanism behind the overexpression of *FRAT1* in ovarian serous adenocarcinomas and its significance is yet to be investigated.

## Figures and Tables

**Table 1:** Association of *FRAT1* and *β*-catenin expression with histological type in ovarian adenocarcinoma

	***FRAT1*, *n* (%)**			***β*-catenin, *n* (%)**	
**Histological type**	**No.**	**High level**	**Low level**	**Accumulation**	**No accumulation**
Serous	41	19 (46)	22 (54)	24 (59)	17 (41)
Clear cell	11	4 (36)	7 (64)	4 (36)	7 (64)
Endometrioid	5	4 (80)	1 (20)	5 (100)	0 (0)
Mucinous	3	1 (33)	2 (67)	0 (0)	3 (100)
					
Total	60	28 (47)	32 (53)	33 (55)	27 (45)

**Table 2:** Comparison of *FRAT1* and *β*-catenin expression in serous adenocarcinoma of ovary

	***FRAT1* expression**		
***β-*Catenin staining**	**Low level (*n*=22)**	**High level (*n*=19)**	**Total**
Accumulation	7 (17%)	17 (41%)	24 (59%)
No accumulation	15 (37%)	2 (5%)	17 (41%)
			
Total	22 (54%)	19 (46%)	41 (100%)

The level of *FRAT1* expression and localisation of *β*-catenin were determined in 41 surgical specimens of serous adenocarcinoma of ovary, as shown in Figure 1. The correlation was analysed using a Spearman rank correlation test, *P*<0.01.
